# Case Report: Hyperinsulinemic hypoglycemia and arterial aneurysms in KAT6B-related disorder: case observation and literature perspective

**DOI:** 10.3389/fendo.2026.1938759

**Published:** 2026-10-08

**Authors:** Nicola Improda, Francesco Maria Rosanio, Giada Ballarin, Fabio Acquaviva, Giangiacomo di Nardo, Lorenza Lepore, Antonia Pascarella, Virginia Mirra, Maria Rosaria Licenziati

**Affiliations:** 1 Neuro-Endocrine Diseases and Obesity Unit, Department of Neurosciences, Santobono-Pausilipon Pediatric Hospital, IRCCS, Naples, Italy; 2 Chronic Diseases, Hepatology and Nutrition Unit, Santobono-Pausilipon Pediatric Hospital, IRCCS, Naples, Italy; 3 Medical Genetics Unit, Department of General and Emergency Pediatrics, Santobono-Pausilipon Pediatric Hospital, IRCCS, Naples, Italy; 4 Cardiology Unit, Santobono-Pausilipon Pediatric Hospital, IRCCS, Naples, Italy; 5 Neonatal Intensive Care Unit, Santobono-Pausilipon Pediatric Hospital, IRCCS, Naples, Italy

**Keywords:** ambiguous genitalia, arterial aneurysms, congenital hyperinsulinism, hypoglycemia, KAT6B-related disorders

## Abstract

**Introduction:**

*KAT6B*-related disorders (*KAT6B*-RD) are rare autosomal dominant chromatinopathies characterized by variable multisystem involvement and marked phenotypic heterogeneity.

**Case presentation:**

We describe a preterm male infant with intrauterine growth restriction who developed recurrent hypoglycemia toward the end of the first week of life during parenteral nutrition weaning, requiring a glucose infusion rate of up to 12 mg/kg/min. Biochemical findings were consistent with hyperinsulinemic hypoglycemia, and glycemic control was rapidly achieved with diazoxide (initiated at 5 mg/kg/day and increased to 6.9 mg/kg/day). After treatment discontinuation at 4 months of age, he exhibited recurrent hypoglycemia again, requiring the resumption of diazoxide. The patient also exhibited complete scrotal agenesis with complex genital anomalies, agenesis of the corpus callosum, generalized hypotonia, severe feeding difficulties requiring gastrostomy, primary hypothyroidism, and aneurysmal dilation of the ascending aorta and proximal right coronary artery. Clinical exome sequencing identified a *de novo* heterozygous pathogenic *KAT6B* variant (c.3995del; p.Pro1332Leufs*2) in exon 17. Diazoxide was successfully discontinued at 12 months of age under continuous glucose monitoring.

**Conclusion:**

This case highlights congenital hyperinsulinism and arterial aneurysms as a clinical association observed in a patient with *KAT6B*-RD. Rather than representing definitive novel features of the syndrome, these manifestations should be interpreted with caution as hypothesis-generating findings potentially linked to *KAT6B*-mediated epigenetic dysregulation, while accounting for potential perinatal confounding factors. These findings nonetheless support careful endocrine and cardiovascular surveillance and follow-up in patients with *KAT6B*-RD.

## 1 Introduction

*KAT6B*-related disorders (*KAT6B*-RD) are autosomal dominant conditions with complete penetrance caused by pathogenic variants in *KAT6B*, which encodes Lysine Acetyltransferase 6B, a histone acetyltransferase involved in chromatin remodeling and transcriptional regulation (1).

These disorders include Genitopatellar Syndrome (GPS) and the Say-Barber-Biesecker-Young-Simpson (SBBYSS) variant of Ohdo syndrome, representing a continuum of phenotypes with variable expressivity (1, 2). Intermediate or overlapping phenotypes are increasingly recognized (2).

Typical manifestations include craniofacial dysmorphism, intellectual disability, hypotonia, genital and skeletal abnormalities, especially patellar hypoplasia or agenesis, alongside variable digital, vertebral, and costal defects. Additional features may include congenital heart disease, gastrointestinal malrotation, feeding difficulties, postnatal growth retardation, cleft or high-arched palate, hearing loss, and dental anomalies (1–3).

To date, *KAT6B*-RD has never been reported in association with congenital hyperinsulinism (CHI).

CHI is a rare cause of persistent hypoglycemia in neonates and infants. It is characterized by inappropriate insulin secretion despite low blood glucose, resulting from genetic defects in β-cell regulatory pathways (4–8). Transient hyperinsulinemic hypoglycemia may occur secondary to perinatal stress, such as maternal diabetes, intrauterine growth restriction (IUGR), asphyxia, or drug exposure, typically resolving within weeks (4). The incidence of non-syndromic genetic forms of hyperinsulinemic hypoglycemia is estimated at 1:25, 000–1:45, 000 live births (5). Variants in the *ABCC8* and *KCNJ11* genes encoding the ATP-sensitive potassium (KATP) channel subunits, which account for most cases, result in either focal or diffuse disease (4–8). CHI has also been reported in several syndromes, such as Beckwith–Wiedemann, Sotos, Simpson–Golabi–Behmel, Kabuki, Rubinstein-Taybi (RST) and Turner syndromes, as well as in congenital disorders of glycosylation (8, 9).

Although genital and cardiovascular malformations are frequent features of KAT6B-RD, the spectrum of these anomalies remains far from being fully delineated (1–3).

This case report aims to describe the clinical presentation of a patient with a *KAT6B*-RD featuring transient hyperinsulinemic hypoglycemia, complete scrotal agenesis, and arterial aneurysms, while providing hypothesis-generating insights and practical management considerations to guide clinicians in the multidisciplinary care of these complex patients based on current literature.

## 2 Case description

### 2.1 Patient information

A male infant was born to non-consanguineous parents following a pregnancy conceived by intracytoplasmic sperm injection. Prenatal ultrasonography identified agenesis of the corpus callosum, bilateral pyelectasis, and a ventricular septal defect. He was born by elective cesarean section for breech presentation at 35 + 6 weeks of gestation. Birth weight was 1, 860 g, length 43 cm, and head circumference 30 cm, all below the 3rd percentile for gestational age according to the sex-specific preterm growth charts (10). Apgar scores were 6 and 8 at 1 and 5 minutes, respectively, with the lower 1-minute score mainly reflecting cyanosis and impaired respiratory effort. Immediately after birth, he required positive-pressure ventilation with bag and mask. Based on the initial clinical evaluation and blood gas analysis performed at the primary hospital, therapeutic hypothermia was deemed unnecessary and was not initiated. At 2 hours of life, the infant was transferred to the neonatal intensive care unit at Santobono-Pausilipon Children’s Hospital for cardiorespiratory monitoring, receiving continuous parenteral nutrition combined with intermittent bolus feedings via gavage. He required non-invasive respiratory support during the first three days of life, with progressive resolution of respiratory distress. At the end of the first week of life, any attempt to decrease parenteral glucose delivery led to recurrent episodes of severe hypoglycemia, necessitating a glucose infusion rate of up to 12 mg/kg/min to maintain normoglycemia.

### 2.2 Clinical findings

The patient had dysmorphic features including blepharophimosis, bulbous nasal tip, high-arched palate, micro/retrognathia, elongated fingers, and a complex genital malformation consisting of micropenis, hypospadias, complete scrotal agenesis, and non-palpable gonads (**Figure 1**).

**Figure 1:**
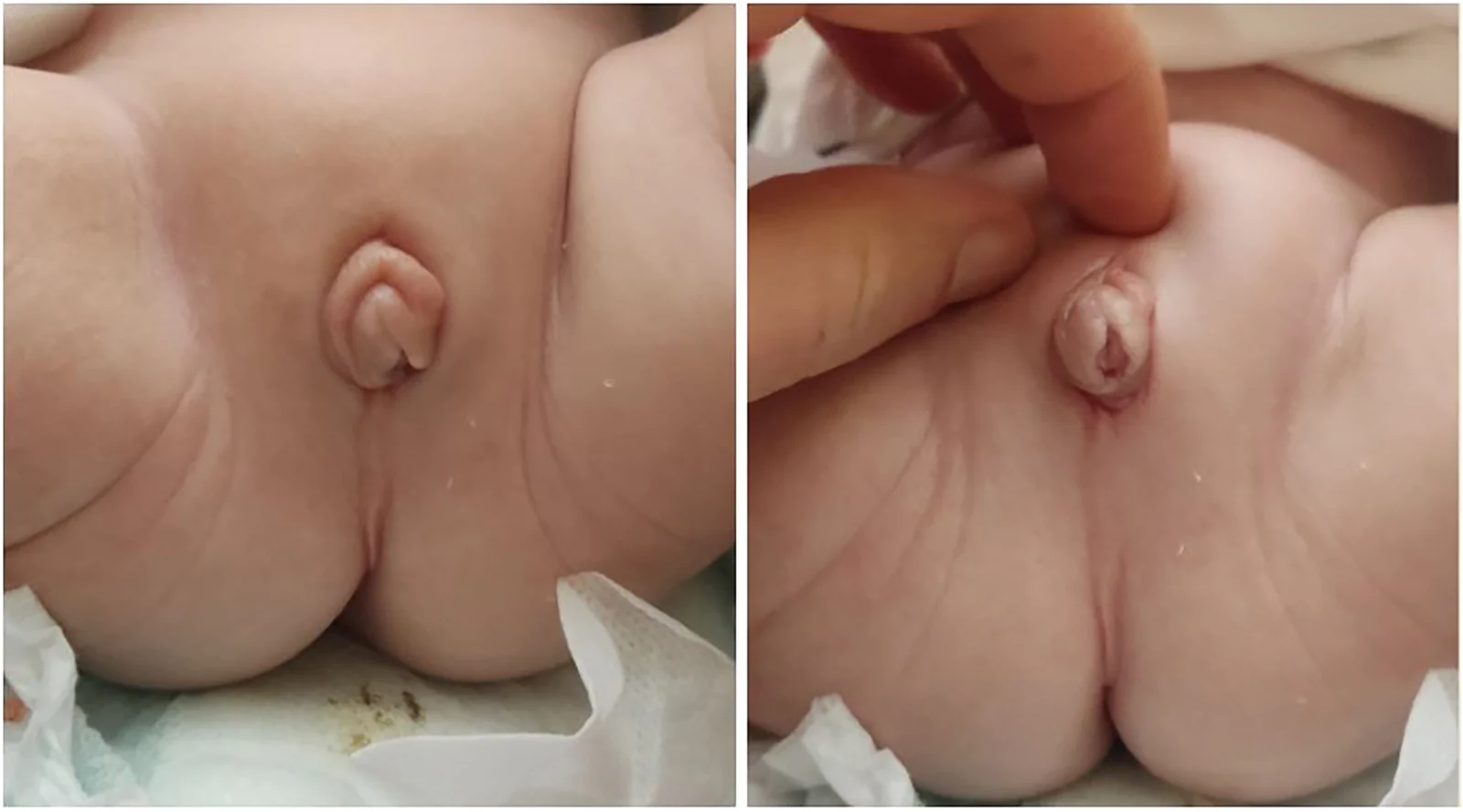
External genital phenotype in the reported patient showing micropenis, proximal hypospadias, complete scrotal agenesis, and bilateral non-palpable gonads. Written informed consent for publication of the image was obtained from the patient’s legal guardians.

He also exhibited truncal hypotonia and severe feeding difficulties requiring percutaneous endoscopic gastrostomy.

Dermatologic features included atopic dermatitis, pityriasis amiantacea, and mild skin hyperelasticity.

### 2.3 Diagnostic assessment

Two critical samples obtained during hypoglycemic episodes showed inappropriately detectable insulin concentrations despite low plasma glucose levels, together with an appropriate counter- regulatory hormonal response and normal ammonia levels, supporting the diagnosis of hyperinsulinemic hypoglycemia (**Table 1**) (4).

**Table 1:** Biochemical and hormonal findings during hypoglycemic critical samples at diagnosis and at 4 months of age.

Parameter	One week of age	Four months of age*	Reference interval	Expected response during hypoglycemia
Blood glucose (mg/dL)	25	34	60-100	Not applicable
Insulin (µIU/mL)	16.05	19.35	1-5	Undetectable/suppressed
C-peptide (ng/mL)	1.0	1.2	0.5-2	<0.5
Cortisol (µg/dL)	13.27	31.5	5-15	>10
Growth hormone (ng/mL)	10.33	10.85	0.05-7.8	>5
Ammonia (µg/dL)	65	60	40-80	<80
Lactate (mmol/L)	2.3	3.1	0.5-2.2	<2.2
Acylcarnitine profile	Normal	Normal	Not applicable	Normal
Plasma amino acids	Normal	Normal	Not applicable	Normal

*One week after diazoxide discontinuation.

Endocrine work-up at three weeks of age showed hormonal values consistent with minipuberty (LH 5.2 mIU/mL, FSH 2.34 mIU/mL, testosterone 1.98 ng/mL) and normal adrenal function, with 17-hydroxyprogesterone 5.3 ng/mL (reference interval [RI], 0.87–6.3 ng/mL), DHEA-S 18.37 μg/dL (RI, 13–125 μg/dL), and androstenedione 0.26 ng/mL (RI, 0.1–1.4 ng/mL), alongside primary hypothyroidism (TSH 66.2 μIU/mL; FT4 0.89 ng/dL, [RI] 0.9–1.7 ng/mL; FT3 within the normal range; negative thyroid antibodies).

Given the abnormal genital phenotype, a standard hCG stimulation test was performed using 1, 000 IU daily for three consecutive days and demonstrated a robust testosterone response, increasing from 0.38 to 6.22 ng/mL. Androstenedione remained within the age-appropriate range after stimulation (0.49 ng/mL), with an androstenedione-to-testosterone ratio of 0.08, while DHEA-S showed only a marginal increase from 18.37 to 27.13 μg/dL. These findings indicated preserved Leydig cell biosynthetic capacity. Karyotype was 46, XY.

Thyroid ultrasound was normal. Abdominal ultrasound identified bilateral intra-abdominal testes near the internal inguinal rings, as well as early nephrocalcinosis with bilateral pyelectasis (6.4 mm on the right and 8 mm on the left; RI <5 mm). Cystourethrography ruled out vesicoureteral reflux.

Cardiac ultrasound revealed a 5-millimeter secundum atrial septal defect with a left-to-right shunt, dysplastic atrioventricular valves, aneurysmal dilation of the ascending aorta (17 millimeters, z-score +4.76), and enlargement of the proximal right coronary artery (2.8 millimeters, z-score +4), all of which were subsequently confirmed by angio-computed tomography and closely monitored through serial echocardiography. The prenatal suspicion of a ventricular septal defect was no longer observed postnatally, likely due to spontaneous intrauterine closure or fetal imaging limitations.

Brain and spine magnetic resonance imaging revealed mild ventriculomegaly (left atrium 11 mm, right 7 mm; RI <10 mm), agenesis of the corpus callosum, hypoplastic olfactory nerves, and dysmorphic ocular bulbs with normal optic nerves. Limb radiographs were unremarkable. Auditory brainstem responses were normal on the left, whereas on the right wave V was elicited at 40 dB nHL by air conduction and 20 dB nHL by bone conduction, consistent with mild-to-moderate conductive hearing loss.

To investigate a genetic etiology for hyperinsulinemic hypoglycemia, targeted next-generation sequencing was performed using a 161-gene panel dedicated to congenital metabolic disorders of carbohydrate and fatty acids. This encompassed genes associated with both isolated and syndromic CHI, including *ABCC8, KCNJ11, GLUD1, GCK, HADH, UCP2, SLC16A1, HNF4A, HNF1A, HK1, PMM2, PGM1, CACNA1D, FOXA2*, and *EIF2S3* (5–7). No pathogenic or likely pathogenic variants in these genes were identified. Because the negative targeted genetic work-up for congenital hyperinsulinemic hypoglycemia failed to account for the patient’s broader syndromic features, clinical exome sequencing (CES) was performed. Genomic DNA extracted from peripheral blood was analyzed using the SOPHiA Genetics Clinical Exome Solution on an Illumina MiSeq platform, with data processed via the SOPHiA DDM v4 bioinformatics platform. The assay covered the coding regions and exon–intron boundaries (± 25 base pair) of more than 4, 900 clinically relevant genes, filtering for regions with a minimum sequencing depth of 30×. The analysis identified a pathogenic heterozygous frameshift variant in *KAT6B* (NM_012330.1:c.3995del; p.Pro1332Leufs*2), which was classified as pathogenic according to the American College of Medical Genetics and Genomics guidelines (11). This variant causes a complete loss of function in a disease-associated gene that exhibits extreme intolerance to genetic variation (observed-to-expected score of 0.128) and is predicted to disrupt critical functional domains. Furthermore, the variant is exceedingly rare in general population databases and has been previously documented in affected individuals (ClinVar Variation ID: 1065489).

### 2.4 Therapeutic intervention

Therapeutic management required a multidisciplinary approach involving neonatology, endocrinology, cardiology, nutrition, and respiratory care.

During the first few days of life, overall fluid intake ranged between 80 and 120 mL/kg/day because of cardiorespiratory instability, with glucose delivery rates of up to 12 mg/kg/min. After the diagnosis of hyperinsulinemic hypoglycemia was established, diazoxide was initiated at 5 mg/kg/day and progressively increased to 6.9 mg/kg/day, successfully achieving stabilization of the glycemic profile.

Following the initiation of diazoxide, the patient developed fluid retention and arterial hypertension. Fluid overload was initially managed with hydrochlorothiazide to counteract diazoxide-related sodium and water retention, resulting in adequate fluid balance and preserved urine output (approximately 4.2 mL/kg/h). Once hydrochlorothiazide was successfully withdrawn following the normalization of fluid status, persistent arterial hypertension was subsequently treated with captopril, which was well tolerated. Renal function remained preserved throughout the hospital stay, with serum creatinine values consistently within the normal range for age.

Daily fluid intake was adjusted according to postnatal age requirements, without the need for additional fluid restriction related to the aneurysmal dilatation. Parenteral nutrition was administered for a total of 31 days and was then gradually discontinued. Subsequently, hydrochlorothiazide could also be safely withdrawn, as the infant tolerated enteral fluid volumes of up to 170 mL/kg/day of fortified formula milk without clinical signs of volume overload.

Because oral feeding remained insufficient to meet nutritional requirements, a percutaneous endoscopic gastrostomy (PEG) was placed. Enteral nutrition was provided through daytime bolus feeds and continuous overnight feeding using a high-energy formula providing approximately 1 kcal/mL.

Primary hypothyroidism was treated with levothyroxine replacement therapy, leading to the normalization of the hormonal profile one month after starting treatment (TSH 1.93 mIU/mL [RI, 0.7–10]; FT3 3.90 pg/mL [RI, 2.10–5.8]; FT4 1.38 ng/dL [RI, 0.89–2.20]).

Severe obstructive sleep apnea was managed with night-time high-flow nasal cannula therapy.

### 2.5 Follow-up and outcomes

At 4 months of age, diazoxide was discontinued; however, one week later, hypoglycemia recurred (plasma glucose 34 mg/dL), with detectable insulin (19.35 μU/mL), C-peptide 1.2 ng/mL, and absent urinary ketones, demonstrating persistence of hyperinsulinemic hypoglycemia beyond the neonatal period (**Table 1**). Diazoxide was therefore restarted.

PET imaging was considered unnecessary because sustained responsiveness to low-dose diazoxide and the absence of pathogenic variants in KATP-channel genes made focal disease highly unlikely according to current international guidelines (8).

At the age of 11 months, anthropometry showed weight 7.4 kg (−1.9 SDS), length 71.5 cm (−1.1 SDS), and head circumference 42 cm (−3.7 SDS). Moreover, he had not achieved independent sitting.

Diazoxide was successfully discontinued at 12 months of age, a process monitored using the continuous glucose monitoring system (FreeStyle Libre 3 Plus, Abbott), which documented a persistently stable glucose profile and fasting tolerance exceeding 8 hours following treatment withdrawal.

The chronological course of the patient’s clinical presentation, diagnostic work-up, therapeutic interventions, and follow-up is summarized in the clinical timeline (**Table 2**).

**Table 2:** Clinical timeline of the patient.

Age/time	Clinical findings	Investigations	Management/outcome
Prenatal	Pregnancy conceived by ICSI; maternal hypertension and Factor V Leiden positivity	Fetal ultrasound: ventricular septal defect, bilateral pyelectasis, and agenesis of the corpus callosum	Pregnancy monitored; delivery planned
Birth (35 + 6 weeks GA)	Preterm male; BW 1, 860 g, length 43 cm, HC 30 cm, all <3rd percentile for GA according to Fenton 2025 growth charts; Apgar scores 6/8 at 1/5 min, with cyanosis and impaired respiratory effort Dysmorphic features, micropenis, hypospadias, complete scrotal agenesis, and non-palpable gonads	Initial postnatal clinical assessment; blood gas analysis performed at the birth hospital Syndromic disorder suspected	Positive-pressure ventilation with bag and mask
First days of life	Transient respiratory distress requiring minimal respiratory support Recurrent hypoglycemia during reduction of parenteral glucose administration; GIR up to 12 mg/kg/min	Cardiorespiratory monitoring. Two critical samples consistent with hyperinsulinemic hypoglycemia; targeted NGS panel for CHI negative for pathogenic/likely pathogenic variants	Progressive resolution of respiratory distress. Diazoxide started at 5 mg/kg/day, with rapid normalization of glycemia Levothyroxine started
First four months of life	Endocrine abnormalities: Hormonal profile consistent with normal minipuberty; normal adrenal function; primary hypothyroidism DSD work-up: 46, XY karyotype; abdominal US: bilateral intra-abdominal testes; hCG stimulation test: preserved testosterone response	Comprehensive multidisciplinary clinical, biochemical, instrumental, and genetic evaluations Clinical exome sequencing identified heterozygous pathogenic KAT6B variant c.3995del (p.Pro1332Leufs*2)	Conservative management and multidisciplinary follow-up Serial echocardiographic surveillance; antihypertensive therapy
	Cardiovascular: ASD, dysplastic AV valves, ascending aortic aneurysmal dilation, and proximal RCA dilation Neurological: Brain MRI: agenesis of the corpus callosum, ventriculomegaly, and hypoplastic olfactory nerves Nutritional: Severe feeding difficulties		Neurological follow-up PEG placement for enteral nutrition Molecular diagnosis of KAT6B-related disorder
4 months	Recurrence of hypoglycemia one week after diazoxide withdrawal	Critical sample: glucose 34 mg/dL, detectable insulin and C-peptide, absent urinary ketones, confirming hyperinsulinemic hypoglycemia	Diazoxide restarted
12 months	Developmental delay; growth retardation	CGM documented normal glucose profile, fasting tolerance >8 h	Multidisciplinary follow-up and rehabilitation. Diazoxide successfully discontinued

ASD, Atrial Septal Defect; BW, Birth Weight; CGM, Continuous Glucose Monitoring; GA, Gestational Age; GIR, Glucose Infusion Rate; HC, Head Circumference; hCG, human Chorionic Gonadotropin; ICSI, Intracytoplasmic Sperm Injection; MRI, Magnetic Resonance Imaging; NGS, Next-Generation Sequencing; NICU, Neonatal Intensive Care Unit; PEG, Percutaneous Endoscopic Gastrostomy; RCA, Right Coronary Artery; US, Ultrasound.

## 3 Discussion

We describe an infant with *KAT6B*-RD in association with diazoxide-responsive hyperinsulinemic hypoglycemia, complete scrotal agenesis, and aortic and coronary aneurysmal dilatations. Given the extreme rarity of these conditions (prevalence is less than one in a million) (1–3), documenting these concurrent clinical features is crucial to assist clinicians in the management of future cases. Rather than redefining the core phenotype, this report aims to document these rare co-occurring manifestations and raise awareness among physicians regarding potentially life-threatening complications in infants presenting with features of *KAT6B*-RD.

*KAT6B* mutations are known to cause two overlapping entities, GPS and SBBYSS, characterized by variable combinations of major and minor anomalies (1).

GPS typically features genital anomalies (clitoromegaly or labia hypoplasia in females; cryptorchidism and scrotal hypoplasia in males), patellar agenesis, flexion contractures, agenesis of the corpus callosum with microcephaly, and renal cysts. SBBYSS presents with blepharophimosis, ptosis, mask-like face, long thumbs/great toes, lacrimal duct anomalies, and patellar hypoplasia/agenesis (1).

Minor features across both syndromes include congenital heart defects, dental anomalies, hearing loss, thyroid anomalies, hypotonia, and global developmental delay, while GPS specifically notes anal anomalies and SBBYSS includes cleft palate and cryptorchidism (1).

The presence of two major features or one major and two minor features suggests a *KAT6B*-RD; however, about 15% of the subjects have an intermediate phenotype between these conditions (1).

Genotype–phenotype correlations have been described in *KAT6B*-RD, with GPS-associated truncating variants generally occurring more proximally in the terminal coding region and SBBYSS-associated variants more frequently affecting distal regions. However, substantial clinical and molecular overlap exists, and variants outside these canonical regions may result in intermediate or overlapping phenotypes (1, 12). Our patient displayed clinical features overlapping both GPS and SBBYSS, supporting classification within the broader *KAT6B*-RD spectrum rather than assignment to a single classical phenotype (**Table 3**). Hence, our case contributes to underlining the questionable validity of diagnostic criteria for specific *KAT6B*-RD, supporting the use of a broader label like *KAT6B*-RD.

**Table 3:** Comparative summary of clinical features in Say–Barber–Biesecker–Young–Simpson syndrome (SBBYSS), Genitopatellar syndrome (GPS), and the present patient.

System/organ	SBBYSS	GPS	Present case	
Cardiovascular	CHD (~50% of affected individuals): ASD, VSD, patent foramen ovale, PDA, aortic arch vessel branching anomaly, supraventricular tachycardia	Same as SBBYSS, but more frequent (up to 67% of affected individuals).	ASD, dysplastic atrioventricular valves, aneurysmal dilation of the ascending aorta and proximal right coronary artery, arterial hypertension	
Craniofacial	Mask-like facies, bulbous nasal tip, icro/retrognathia, thin upper lip, blepharophimosis, Pierre-Robin sequence, cleft/high-arched palate, dacryostenosis, dolichocephaly, sagittal craniosynostosis	Less distinctive face, cleft palate, micro/retrognathia, dolichocephaly, broad nasal bridge, scaphocephaly	Blepharophimosis, high-arched palate, micro-/retrognathia	
Endocrine	Primary hypothyroidism (rarely thyroid agenesis or hypoplasia), GH deficiency, post-natal growth restriction, delayed bone age	Same as SBBYSS but often more severe endocrine involvement, delayed puberty	IUGR, congenital primary hypothyroidism, diazoxide-responsive hyperinsulinemic hypoglycemia	
Eyes	Blepharophimosis, ptosis strabismus, amblyopia, optic atrophy, nystagmus, hypertelorism	Same as SBBYSS impairments in smooth pursuit eye movements	Dysmorphic ocular bulbs, normal optic nerves	
Gastrointestinal	Feeding difficulties, GERD, constipation, malrotation, anus imperforate	Feeding difficulties, GERD, diaphragmatic eventration, anal atresia/stenosis, rectal duplication, anteriorly positioned anus	Feeding difficulties requiring PEG	
Respiratory	Laryngomalacia, apnea, sleep apnea, tracheomalacia	Often severe respiratory distress, pulmonary hypoplasia	Neonatal respiratory distress, mild obstructive sleep apnea	
Genitourinary	Cryptorchidism, ambiguous genitalia, bicornuate uterus (rare), vesicoureteral reflux	Micropenis, cryptorchidism, scrotal hypoplasia, clitoromegaly and/or hypoplasia of the labia minora or majora, renal anomalies	Micropenis, hypospadias, cryptorchidism, scrotal agenesis, nephrocalcinosis, renal pelvis dilation	
Hearing	Sensorineural or conductive loss, narrowing of the ear canal	Same as SBBYSS	Conductive hearing loss
Musculoskeletal	Long thumbs/toes, polydactyly, scoliosis, hypermobility, pectus deformities	Patellar agenesis, contractures, thoracic/pelvic anomalies	Elongated fingers
Nervous system	Global developmental delay, intellectual disability, behavioral and/or psychiatric issues, neonatal hypotonia, seizures(rare), hypoplasia of the corpus callosum (rare), ventriculomegaly, pachygyria, myelination delay, gliosis, lissencephaly	Global developmental delay, intellectual disability, behavioral and/or psychiatric issues, neonatal hypotonia, seizures, agenesis or hypoplasia of the corpus callosum (nearly all), cortical malformations, hydrocephaly, ventriculomegaly	Global developmental delay, agenesis of corpus callosum, olfactory nerve hypoplasia
Skin/Nails	Sparse hair, dry skin, hypoplastic nails	Hemangiomas, café-au-lait spots, redundant skin	Atopic dermatitis, pityriasis amiantacea of the scalp, mild hyperelasticity

ASD, Atrial Septal Defect; CHD, Congenital Heart Defects; CNS, Central Nervous System; GERD, Gastroesophageal Reflux Disease; GH, growth hormone; GPS, Genitopatellar Syndrome; IUGR, Intrauterine Growth Restriction; PDA, patent ductus arteriosum; PEG, Percutaneous Endoscopic Gastrostomy; SBBYSS, Say-Barber-Biesecker-Young-Simpson Syndrome; VSD, ventricular septal defect.

Endocrine abnormalities have also been reported within the *KAT6B*-RD spectrum, including congenital primary hypothyroidism, which was observed in our patient. Although primary hypothyroidism in this context can stem from altered glandular development (1), our patient’s thyroid ultrasound showed no overt structural abnormalities. This is consistent with data from previous reports (3).

In contrast, hyperinsulinemic hypoglycemia has not been reported in association with *KAT6B*-RD. CHI is the leading cause of persistent hypoglycemia in early life and can cause irreversible neurological damage if unrecognized (5–8). In some cases, it is reported in the context of overgrowth syndromes (i.e. Beckwith-Wiedemann, Sotos, and Simpson-Golabi-Behmel syndromes), or developmental syndromes, such as Kabuki, RST and Turner syndromes, and certain congenital disorders of glycosylation (8, 9). However, the spectrum of disorders associated with CHI is expanding over time, even if the underlying pathogenic mechanism remains unproven in certain cases (7, 9, 13).

Although deep intronic pathogenic variants in established CHI genes cannot be definitively excluded due to sequencing limitations, the occurrence of hyperinsulinemic hypoglycemia in the context of *KAT6B*-RD raises intriguing questions regarding the underlying pathogenic mechanisms.

Perinatal stress-induced hyperinsulinism represents a likely explanation for the hyperinsulinemic phenotype (8). Our patient was born preterm and SGA following a pregnancy complicated by fetal growth restriction and required respiratory support at birth, which all represent relevant potential factors causing transient hyperinsulinemic hypoglycemia. Indeed, the recurrence of hyperinsulinemic hypoglycemia following diazoxide withdrawal at 4 months, alongside its eventual permanent discontinuation at 12 months is also consistent with a prolonged transient etiology.

Secondarily, the co-occurrence of *KAT6B*-RD and hyperinsulinemic hypoglycemia draws interesting biological parallels with other chromatin-remodeling conditions. Indeed, lysine Acetyltransferase 6B, the histone acetyltransferase (HAT) encoded by *KAT6B*, participates in multiprotein complexes involved in chromatin remodeling and transcriptional regulation, influencing key cellular processes such as cell-cycle control, differentiation, and cellular responses to stress (14–16). By analogy with what has been hypothesized in Kabuki syndrome (17), it is conceivable that in patients with *KAT6B*-RD, CHI might be caused by aberrant epigenetic regulation of genes involved in β-cell development or function. This hypothesis gains further support when considering RST syndrome, another disorder linked to epigenetic regulators, where hyperinsulinism is specifically associated with the EP300 variant, accounting for 8% of all molecularly confirmed RST cases (9). Indeed, EP300 is functionally similar to *KAT6B*, as it also encodes a HAT protein and has been shown to be required, along with CREBBP, for normal pancreatic islet cell development, beta-cell function, and survival (9). Notably, studies in mouse models lacking EP300 or CREBBP in pancreatic islet cells exhibit hypoinsulinemia, which stands in contrast to the hyperinsulinism observed in human cases (18). This discrepancy highlights the complexity of HAT-mediated regulation in beta-cell function and underscores that the observed association between the *KAT6B* variant and hyperinsulinism is strictly hypothesis-generating, warranting further mechanistic studies to determine the role of epigenetic dysregulation.

Severe genital malformations, including cryptorchidism, micropenis, hypospadias, and penoscrotal transposition, are well documented in *KAT6B*-RD (19). Our case further broadens this spectrum by demonstrating complete scrotal agenesis (**Figure 1**). Notably, this feature leads to ambiguous genitalia, posing challenges to sex assignment at birth, especially where karyotype analysis is not readily available or the gonads are non-palpable (20).

In addition, we provide further evidence of an intact testicular endocrine function during infancy in *KAT6B*-RD (20), by integrating baseline data with hCG-stimulated testosterone concentrations.

These findings suggest that the genital abnormalities are unlikely to result from a primary defect in steroid hormone biosynthesis. Notably, functional studies in *KAT6B*-RD patients have shown reduced histone H3 and H4 acetylation (21), reflecting impaired HAT activity. Because KAT6B acts as a transcriptional co-activator, this epigenetic dysregulation likely disrupts tissue-specific developmental programs, including those involved in genital organogenesis where *KAT6B* is expressed (22). While direct epigenetic profiling of genital tissues remains challenging, these mechanistic and expression data support this hypothesis, though the specific downstream pathways require further investigation. Our data are consistent with most published cohorts reporting normal pubertal development in *KAT6B*-RD. However, it is worth noting that delayed puberty has been described, ranging from mild delay in a male with SBBYSS to severe delay in a female with GPS (19, 23). In particular, this latter case described a gonadotropin rise up to the pubertal range after the age of 15 years, despite lack of secondary sex characteristics at 18 years of age (19). Collectively, these data suggest that variable degrees of gonadal dysfunction may occasionally occur in *KAT6B*-RD, possibly depending on the genotype (23).

Finally, another notable clinical finding observed in our patient was the aneurysmal dilation of the ascending aorta and proximal right coronary artery. Interestingly, Han et al. reported significantly increased *KAT6B* expression in human abdominal aortic aneurysm tissue compared with healthy aortae, at both the mRNA (2.8-fold) and protein (2.2-fold) levels, with expression levels significantly associated with T-cell infiltration within the aneurysmal wall (24). While these findings highlight a potential link between altered *KAT6B* expression and the inflammatory and epigenetic milieu of acquired abdominal aortic aneurysms, they derive from adult disease and provide only indirect biological evidence. Given the patient’s mild skin hyperelasticity, major hereditary aortopathy-associated genes—including *FBN1, TGFBR1, TGFBR2, SMAD3*, and *COL3A1*—were evaluated through the CES data, yielding no pathogenic or likely pathogenic variants. While high-throughput sequencing data alone cannot establish causality, the systematic exclusion of these primary syndromic aortopathies helps mitigate alternative genetic diagnoses. Although this vascular manifestation may represent a rare co-occurring association in the context of *KAT6B*-RD, its direct pathogenetic relationship remains speculative and requires careful validation in future cohorts and functional studies.

## 4 Strengths and limitations

The strengths of this report include detailed endocrine, genetic, cardiovascular, and neuroradiological characterization, longitudinal follow-up, and objective glucose assessment by continuous glucose monitoring during diazoxide withdrawal. The main limitations of this study include the single-patient design and the absence of functional studies to establish whether a direct pathogenetic link underlies the rare clinical associations reported. Furthermore, although rare, deep intronic pathogenic variants causative of the observed comorbidities cannot be entirely excluded by the applied genetic methodologies.

## 5 Patient perspective

The patient’s parents reported that establishing a molecular diagnosis substantially improved their understanding of their child’s condition and facilitated long-term multidisciplinary care planning.

They described diazoxide therapy as highly effective in preventing hypoglycemic episodes and considered the coordinated management provided by the multidisciplinary team essential for their child’s clinical stability. They also reported that the use of continuous glucose monitoring during diazoxide withdrawal provided reassurance by allowing close monitoring of glucose levels at home, reducing anxiety about recurrent hypoglycemia and increasing confidence in the therapeutic strategy.

Overall, they perceived a significant improvement in disease management and quality of life following diagnosis and treatment.

## 6 Conclusions

This report describes the rare co-occurrence of transient hyperinsulinemic hypoglycemia, complete scrotal agenesis, and arterial aneurysms in a patient with *KAT6B*-RD. In addition, it provides valuable clinical documentation regarding preserved testicular function in this condition.

While *KAT6B*-dependent epigenetic dysregulation is a plausible hypothesis, further well-characterized cases and functional studies are needed to determine whether hyperinsulinemic hypoglycemia and arterial aneurysms represent recurrent manifestations within the *KAT6B*-RD spectrum.

Ultimately, our case confirms the complex presentation of *KAT6B*-RD—where genetic diagnosis is increasingly facilitated by next-generation sequencing—and highlights the critical need for a coordinated, multidisciplinary management approach.

## Data Availability

The raw data supporting the conclusions of this article will be made available by the authors, without undue reservation.
